# Glycoprotein Targeted CAR-NK Cells for the Treatment of SARS-CoV-2 Infection

**DOI:** 10.3389/fimmu.2021.763460

**Published:** 2021-12-23

**Authors:** Ilias Christodoulou, Ruyan Rahnama, Jonas W. Ravich, Jaesung Seo, Sergey N. Zolov, Andrew N. Marple, David M. Markovitz, Challice L. Bonifant

**Affiliations:** ^1^ The Sidney Kimmel Comprehensive Cancer Center, Johns Hopkins University School of Medicine, Baltimore, MD, United States; ^2^ Department of Pediatrics, Johns Hopkins University School of Medicine, Baltimore, MD, United States; ^3^ Department of Internal Medicine, University of Michigan, Ann Arbor, MI, United States; ^4^ Department of Medicine, Johns Hopkins University School of Medicine, Baltimore, MD, United States; ^5^ Division of Infectious Diseases, Department of Internal Medicine, and the Programs in Immunology, Cellular and Molecular Biology, and Cancer Biology, University of Michigan, Ann Arbor, MI, United States

**Keywords:** SARS-CoV-2, CAR-NK cells, immunotherapy, glycobiology, Banana Lectin, COVID-19

## Abstract

H84T-Banana Lectin (BanLec) CAR-NK cells bind high mannose glycosites that decorate the SARS-CoV-2 envelope, thereby decreasing cellular infection in a model of SARS-CoV-2. H84T-BanLec CAR-NK cells are innate effector cells, activated by virus. This novel cellular agent is a promising therapeutic, capable of clearing circulating SARS-CoV-2 virus and infected cells. Banana Lectin (BanLec) binds high mannose glycans on viral envelopes, exerting an anti-viral effect. A point mutation (H84T) divorces BanLec mitogenicity from antiviral activity. SARS-CoV-2 contains high mannose glycosites in proximity to the receptor binding domain of the envelope Spike (S) protein. We designed a chimeric antigen receptor (CAR) that incorporates H84T-BanLec as the extracellular moiety. Our H84T-BanLec CAR was devised to specifically direct NK cell binding of SARS-CoV-2 envelope glycosites to promote viral clearance. The H84T-BanLec CAR was stably expressed at high density on primary human NK cells during two weeks of ex vivo expansion. H84T-BanLec CAR-NK cells reduced S-protein pseudotyped lentiviral infection of 293T cells expressing ACE2, the receptor for SARS-CoV-2. NK cells were activated to secrete inflammatory cytokines when in culture with virally infected cells. H84T-BanLec CAR-NK cells are a promising cell therapy for further testing against wild-type SARS-CoV-2 virus in models of SARS-CoV-2 infection. They may represent a viable off-the-shelf immunotherapy for patients suffering from COVID-19.

## Introduction

The prevalence and pathogenicity of the novel severe acute respiratory syndrome coronavirus 2 (SARS-CoV-2) has caused an international pandemic that has placed healthcare systems under unprecedented stress. Lack of prior exposure and a high fatality rate of coronavirus disease 2019 (COVID-19) (1), the disease caused by SARS-CoV-2 infection, have thus far resulted in almost four million deaths worldwide (as per the World Health Organization). Despite the development and administration of effective vaccines (2, 3), the infection and hospitalization rates due to COVID-19 remain significant, necessitating effective and readily available treatment options. Critically ill patients with COVID-19 have immune dysregulation with reduced number and function of effector cells, which impairs viral clearance (4–11). The degree of observed reduction and phenotypic alteration of B, CD4+ T, CD8+ T, and natural killer (NK) cells correlates with disease severity (5, 8–10). Thus, a strategy to enhance, activate, and repopulate patient immune cells is necessary to better control SARS-CoV-2 infection.

NK cells are innate lymphocytes of the immune system with an important role in the control of viral infections (12, 13). NK cell activation and resultant cytotoxicity is regulated by the interplay of inhibitory and activating receptors (14, 15). NK cells are inhibited by MHC-I binding of Killer Immunoglobulin-like Receptors. Importantly, virally infected cells downregulate MHC-I expression which disallows NK cell inhibition (16, 17). In addition, virus-derived products and stress-induced ligands are expressed on infected cells, which act to stimulate NK cell activating receptors and enhance the strength of signal initiated by loss of inhibition (17, 18). These combined signals ultimately determine the power of NK cell cytotoxicity against virally infected cells (15, 17). To add to this innate activated state, NK cells that express chimeric antigen receptors (CARs) display further antigen-specific activation, providing cells with boosted and directed antiviral potential (19). The functionality of CARs is heavily dependent on target antigen selection (20). Many RNA viruses, including SARS-CoV-2, evade immunological recognition by mutation of key amino acids (21, 22). As the majority of CAR constructs bind targets *via* an extracellular single-chain variable fragment specific to a protein epitope (23), there is a need for the development of CARs with receptor targeting patterns that are less likely to be impacted by genomic point mutations.

The SARS-CoV-2 virus mediates cell entry *via* association of its trimeric spike protein with human Angiotensin-Converting Enzyme 2 (ACE2) (24, 25). The spike protein is decorated with under processed high mannose, as is common in other viruses (26–28). In the case of SARS-CoV-2, virus-specific high mannose N-glycosites are in close proximity to the receptor binding domain (RBD) of the spike protein (26). These glycosylation sites can serve as a targeting pattern for CARs. Banana Lectin (BanLec) is a lectin extracted from the fruit of bananas (Musa acuminate) that binds high mannose glycans (29, 30). The binding of BanLec to virally expressed high mannose glycans has antiviral activity (30, 31), but wild-type BanLec is also strongly mitogenic and induces nonspecific T cell activation (32). BanLec mitogenicity can be divorced from antiviral activity *via* a single amino acid change (H84T) (32). H84T-BanLec retains its binding capacity to HIV, influenza, and coronaviruses (32–34). However, lectins are subject to chemical and biomolecular degradation, making systemic administration challenging (35). Consequently, enhanced durability of H84T-BanLec treatments could potentially improve antiviral efficacy.

We sought to improve H84T-BanLec antiviral activity by combining its exquisite viral targeting and the innate activity of primary NK cells. We designed a H84T-BanLec CAR containing the 4-1BB (CD137) and TCRζ intracellular domains and expressed this artificial chimeric receptor at the NK cell membrane. H84T-BanLec chimeric antigen receptor natural killer (CAR-NK) cells diminished the potency of lentivirus pseudotyped with a SARS-CoV-2 spike protein (S-protein) envelope. Binding of target stimulated NK cell activation, likely driven initially by innate antiviral cell responses, and then boosted by CAR molecular signaling. Glycosylation-directed CAR-NK cells such as H84T-BanLec CAR-NK are activated against SARS-CoV-2 and have the potential to blunt viral infection, potentially through clearance of virally-infected cells or prevention of viral entry.

## Results

### H84T-BanLec.4-1BB.ζ CAR Is Stably Expressed in Human NK Cells

**Figure 1:**
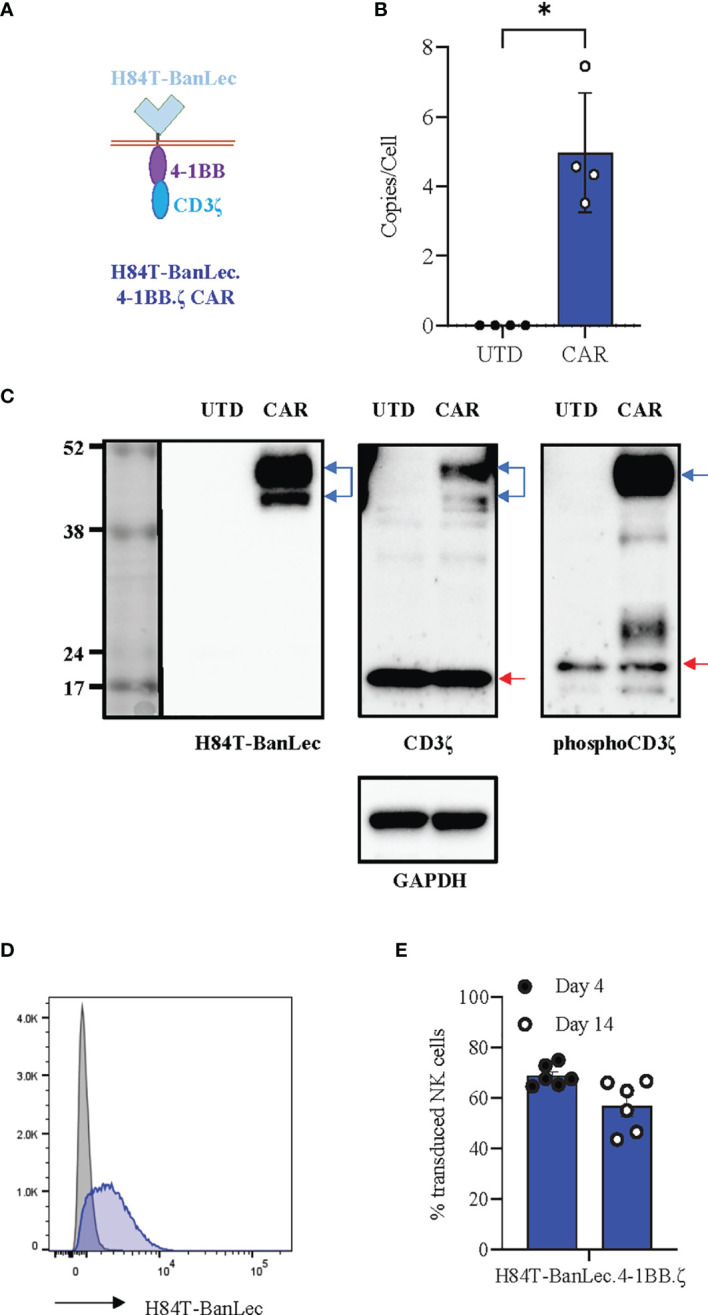
H84T-BanLec.4-1BB.ζ CAR expression in human NK cells. **(A)** Schema defining CAR components. **(B)** Quantification of retroviral vector copy number (VCN) in transduced NK cells (CAR). Untransduced/unmodified (UTD) NK cells served as negative controls. n = 4 NK cell donors. **(C)** Western blot detection of protein. UTD: untransduced NK cell lysate, CAR: H84T-BanLec CAR-NK cell lysate. Blue arrows: CAR, red arrows: endogenous zeta chain. GAPDH was used as a loading control. **(D)** Representative histogram showing detection of CAR-expression with flow cytometry. Gray: UTD, Blue: CAR-NK. **(E)** CAR detection on primary NK cell surface on days 4 and 14 post-transduction. Each dot representative of single transduction. n = 6 total replicates from 4 independent NK cell donors. *p < 0.05.

We synthesized the H84T-banana lectin (H84T-BanLec) (32) sequence and subcloned this in place of the extracellular binding domain of an existing 4-1BB.ζ CAR (36). Our complete CAR was comprised of H84T-BanLec, CD8α hinge and transmembrane domains, and the intracellular domains of 4-1BB and the CD3ζ chain (**Figure 1A**). We produced replication incompetent retrovirus carrying our CAR sequence and used this to generate H84T-BanLec CAR NK cells. We measured a median of 4.5 integrated CAR copies per cell (range 3.5-7.45, **Figure 1B**) and verified expression of CAR protein using Western blot (**Figure 1C**). We observed constitutive CD3ζ phosphorylation of the CAR in our transduced cells, a finding that has previously been associated with CAR tonic signaling (**Figure 1C**) (37, 38). We verified surface expression using flow cytometry and measured stable expression of the H84T-BanLec.4-1BB.ζ CAR on the surface of human NK cells over the tested period of two weeks (day 4 post-transduction: median [range], 67.5% CAR-positive [64.7-75%], day 14 post-transduction: 58.9% CAR-positive [43.6-66.7%], **Figures 1D, E**).

### hACE2-Expressing 293T Cells Bind SARS-CoV-2 Envelope Proteins

**Figure 2:**
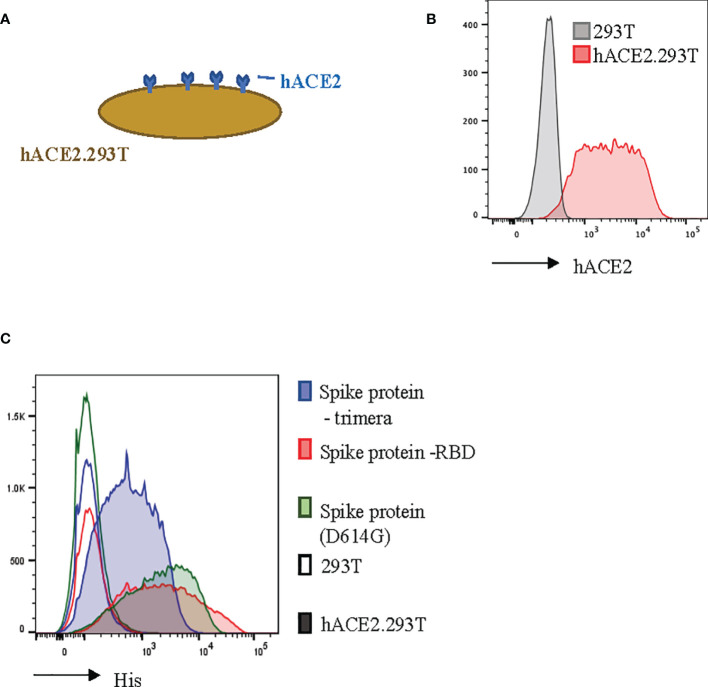
Recombinant SARS-CoV-2 proteins bind hACE2.293T. **(A)** Schema of 293T engineered with hACE2. **(B)** Flow cytometric analysis of 293T expressing hACE2. **(C)** Detection of recombinant SARS-CoV-2 spike (S)-proteins bound to hACE2-expressing 293T cells. 293T without hACE2 expression used as negative control.

In order to model SARS-CoV-2 infection, we engineered 293T cells to constitutively express the ACE2 transmembrane protein (**Figures 2A, B**). Human ACE2 is the binding partner for SARS-CoV-2 (24, 39). We found that our hACE2.293T cells bound trimeric S-protein, the S-protein Receptor Binding Domain (RBD), and the D614G mutated (21) S-protein (**Figure 2C**). Unmodified 293T cells did not bind S-proteins (**Figure 2C**).

### H84T-BanLec CAR-NK Cells Decrease Cellular Pseudovirus Infection

**Figure 3:**
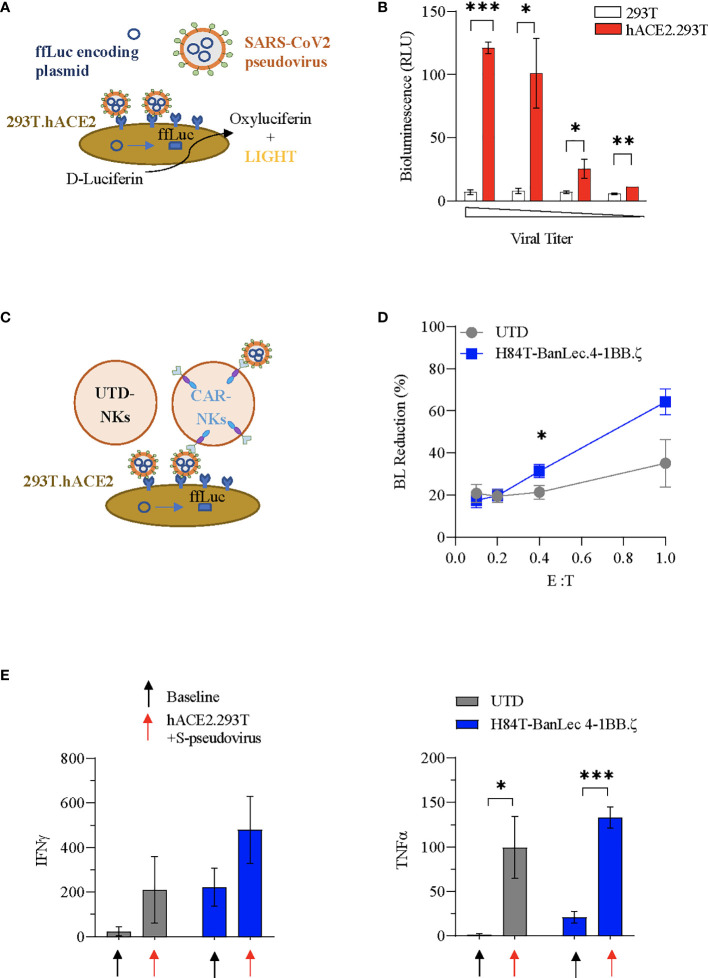
H84T-BanLec CAR-NK cells decrease S-pseudotyped viral infection. **(A)** Schema of SARS-CoV-2 pseudovirus infection of hACE2.293T. Pseudoviral particles contain plasmids encoding ffLuc. Following viral entry, cells emit bioluminescence (BL) after D-Luciferin metabolism. **(B)** Measurement of target cell BL emission following transduction with S-protein pseudotyped virus carrying ffLuc reporter gene. Assay performed in triplicate. **(C)** Schematic representation of the BanLec-CAR NK cells blocking hACE2.293T infection. **(D)** NK cells plated with target cells (hACE2.293T) and pseudovirus at indicated effector to target (E:T) ratios. Percent bioluminescence (BL) reduction from maximal measured on day 2. The condition with 293T.ACE2 and pseudovirus alone (100%) represents the positive control (E:T = 0.4, p = 0.034; E:T = 1, p = 0.054, n = 6, 2 separate experiments using 3 independent NK cell donors, each experiment performed in triplicate). **(E)** Quantification (pg/mL) of IFNγ and TNFα present in culture media of NK cells at baseline and in co-culture with S-pseudotyped virus infected hACE2.293T (black: no target, red: co-culture; n=3 donors; Mean value+/- SEM; baseline vs. co-culture *p < 0.05, **p < 0.01, ***p < 0.001, not significant if not indicated).

We used SARS-CoV-2 envelope pseudotyping of a replication deficient lentiviral vector (40) in order to evaluate whether H84T-BanLec CAR NK cells could mediate clearance of SARS-CoV-2. We first tested hACE2.293T transduction using S-protein pseudotyped lentiviral particles. The pseudotyped vector carried firefly luciferase (ffLuc, **Figure 3A**). Viral entry into cells was verified by quantification of bioluminescence (BL) emission following addition of D-Luciferin to virally transduced cells. Functionality of our assay was confirmed with observed BL emission of transduced hACE2.293T cells at all tested viral titrations. In contrast, 293T without ACE2 expression were not transduced, confirming specificity of viral binding and entry dependent on hACE2 (**Figure 3B**).

We next investigated whether H84T-BanLec CAR-NK cells could specifically target S-protein pseudoviral transduced hACE2.293T. NK cells (CAR-NKs or unmodified) were plated with hACE2.293T and freely circulating pseudoviral particles (**Figure 3C**). We observed a reduction in SARS-CoV-2 pseudoviral mediated bioluminescence emission from hACE2.293T cells when H84T-BanLec CAR-NK cells were present (**Figure 3D**). This reduction, which is indicative of a decrease in the total number of virally infected human cells, was noted in both unmodified and H84T-CAR NK cells, but was more pronounced when CAR-NK cells were present (mean % BL reduction +/- SEM of hACE2.293T in cocultures with unmodified NK vs. H84T-BanLec CAR-NK; 35 +/-11% vs 64%+/- 6% for 1:1 effector-to-target ratio, p=0.054; 21+/-3% vs 31%+/- 3% for 1:2.5 effector-to-target ratio, p=0.034; **Figure 3D**). NK cells are reactive immune effector cells with cytotoxic potential against allogeneic targets. Therefore, we analyzed the killing capacity of NK cells against hACE2.293T cells to determine whether differences seen in the clearance of virally-infected cells could be explained by nonspecific target killing. There were no observed differences between CAR- and unmodified NK cell killing of hACE2.293T cells across a range of effector-to-target (E:T) ratios (mean % cytotoxicity +/- SEM of unmodified NK vs. H84T-BanLec CAR-NK; 23.5 +/-2.7% vs. 25.9+/- 7% in 1:1 E:T ratio, p=0.76; 20.6 +/-2% vs 16.1%+/- 1% in 1:2.5 E:T ratio, p=0.1; **Figure S1**). Moreover, the measured cytotoxicity of NK cells against 293T cells was roughly equivalent to the percent decrease in BL emission noted when unmodified NK cells were used in our pseudoviral infectivity assay. This suggests that background cytotoxicity may have contributed to lowered BL in the absence of H84T-BanLec viral binding.

### H84T-BanLec CAR-NK Cells Are Strongly Activated by Virus

We evaluated the activation of NK cells in our pseudovirus assay. Both unmodified and H84T-BanLec CAR-NK cells were stimulated to secrete inflammatory mediators when co-cultured with pseudoviral particles and virally infected cells, including IFNγ (mean pg/ml +/- SEM of NK cells at baseline vs. in coculture with hACE2.293Tand S-pseudovirus; unmodified NK: 24 +/-11.6 vs. 209.4 +/- 86.1, p=0.16; CAR-NK: 221.9 +/- 49.7 vs. 479.2 +/- 86.7, p=0.07;n=3, **Figure 3E**) and TNFα (unmodified NK: 1.2 +/- 0.6 vs. 99.3 +/- 20.1, p=0.03; CAR-NK: 20.9 +/- 3.6 vs. 132.6 +/- 6.8, p=0.0007; n=3, **Figure 3E**) CAR-NK cells showed overall higher cytokine secretion both at baseline and with viral stimulation.

## Discussion

**Figure 4:**
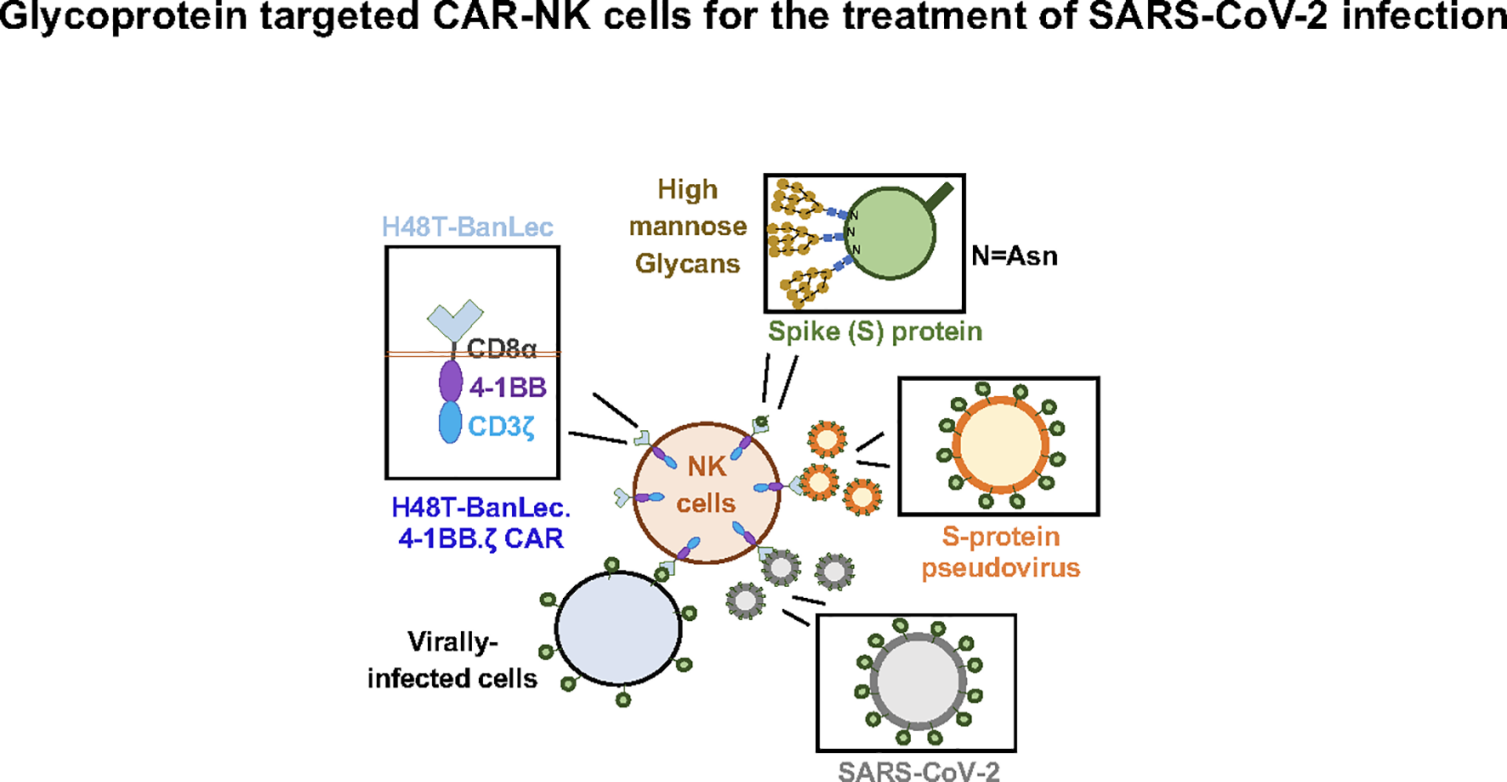
H84T-Banana Lectin (BanLec) CAR-NK cells are activated by binding high mannose glycosites that decorate the SARS-CoV-2 envelope. This binding can diminish S-protein pseudotyped viral infectivity.

In this study we show successful generation of CAR-NK cells expressing extracellular H84T-BanLec linked to intracellular activation domains (**Figure 4**). Surface expression of the lectin-containing CAR was associated with tonic phosphorylation of CAR-CD3ζ, validating its molecular functionality. We also engineered 293T cells to express ACE2, the SARS-CoV-2 receptor protein. We employed a model of SARS-CoV-2 infection that used a lentiviral vector pseudotyped with the SARS-CoV-2 S-protein. H84T-BanLec CAR-NK cells promoted a decrease in the number of total cells infected with pseudovirus after two days of culture. Moreover, our CAR-NK cells increased their secretion of IFNγ and TNFα after encountering virally infected cells. Increased inflammatory cytokine secretion was also observed to a lesser extent in unmodified NK cells, illustrating NK cell innate anti-viral potency.

CAR-T and CAR-NK cells are emerging immunotherapies with great promise. Typically, the expressed synthetic receptors are designed to bind surface protein. However, in our study, we designed a CAR making use of a unique extracellular moiety with binding properties dependent on target glycosylation. Targeting of glycoprotein, and specifically N-glycosylation products, with a CAR is rare. We believe that we describe the first CAR targeting the N-glycans dispersed on SARS-CoV-2 envelope proteins. Furthermore, to our knowledge this is the first lectin-based CAR designed and functionally tested in pre-clinical study. As lectins have evolved over millions of years to be highly potent and selective, the H84T-BanLec CAR represents an entirely new approach in that it targets aberrant glycosylation patterns in viral proteins. This methodology has the potential to be applied against cancer and other target cells as well.

During viral infections NK cells have a central role as first responders (18). Viral infections can activate NK cells to produce IFNγ, TNFα, and other immunity-enhancing mediators that prime the adaptive immune response (13, 18, 41). However, many viruses, including SARS-CoV-2, deploy strategies to evade NK cell surveillance (42, 43). CAR expression on the surface of NK cells can potentiate endogenous and antigen-specific activation and target killing (44). This boosted function may render CAR-NK cells superior effectors in clearing circulating virus and virally infected cells. Appropriate antigen targeting and a precise combination of intracellular signaling domains are critical to direct CAR-NK cell behavior. We and others (44–47) have found 4-1BB and CD3ζ intracellular domains to be a good combination for NK cell activation. The vast majority of CAR constructs contain an extracellular single chain variable fragment derived from a monoclonal antibody for protein binding (23). Instead, we used a lectin with specific binding to high mannose, an altered glycosylation pattern common to viral envelopes (26–30). Our glycoprotein targeting mitigates the potential risk of antigen downregulation, a mechanism commonly employed by cancer cells to evade targeted immunotherapies (48). CAR binding to envelope glycoproteins has the potential to not only neutralize and eliminate circulating virus, but also to clear infected cells with retained envelope proteins on their surface.

We observed decreased BL likely secondary to a combination of viral clearance and specific targeting of virally infected cells by our CAR-NK cells. We also observed stable CAR.ζ phosphorylation and heightened activation of CAR-NK cells when compared to unmodified NK cells. This 4-1BB.ζ induced activation may prevent the dysfunction seen in circulating NK cells of patients with COVID-19 (11, 49), similar to 4-1BB.ζ CAR mitigation of T cell exhaustion (38). Ultimately, investigation of H84T-BanLec CAR-NK cell efficacy against wild-type SARS-CoV-2 virus and in animal models of SARS-CoV-2 infection is needed.

Acute deterioration with COVID-19 requires emergency treatment options available at the ready. In comparison to T cells, which must be manufactured from autologous cells in order to prevent graft-versus-host disease, NK cells can be infused from allogeneic donors without this risk (50, 51). Aliquots of manufactured CAR-NK cells can be frozen and stored to establish a master cell bank capable of treating patients suffering from COVID-19 and in need of adequate immune function. Indeed, there are open clinical trials investigating the potential role of NK cell (NCT04280224, NCT04365101, NCT04634370, NCT04797975, NCT04900454) and CAR-NK cell (NCT04324996) adoptive transfer as COVID-19 treatments.

Infusion of SARS-CoV-2 targeted NK cells has the potential to exacerbate COVID-19-associated hyperinflammatory syndrome and its resultant organ failure (52, 53). Peripheral blood circulating (CD56^hi^) NK cells are decreased during severe COVID-19 (11). This suggests that the NK cells of ill patients are not directly contributory to COVID-19 cytokine storm (11). However, activated CAR-NK cells are capable of producing proinflammatory cytokines that can potently activate macrophages and potentially exacerbate underlying hypercytokinemia and tissue damage. It is encouraging that treatment with H84T-BanLec is safe in murine models (33). However, the risk of unspecific toxicity secondary to NK cell surface H84T-BanLec binding of glycosylated protein on normal tissues should also be tested using tissue arrays and relevant *in vivo* model systems. Overall, it is imperative to identify disease conditions in which this unique therapy has a favorable risk-benefit profile.

Our findings support the hypothesis that CAR-NK cells expressing H84T-BanLec can mediate SARS-CoV-2 viral clearance. Taken together with the innate antiviral activity and capability for allogeneic infusion of NK cells, H84T-BanLec CAR-NK cells exhibit favorable characteristics that support further testing. If found feasible, translation to the clinic could potentially impact days of hospitalization and survival rates of COVID-19 patients.

## Materials and Methods

### Cell Lines

HEK293T cells were purchased from the American Type Culture Collection (ATCC, Manassas, VA) and grown in Dulbecco’s Modified Eagle Medium (DMEM; ThermoFisher Scientific Waltham, MA), supplemented with 10% Fetal Bovine Serum (FBS; HyClone, Logan, UT). High-expressing human ACE2 (hACE2) 293T cells were created by first subcloning hACE2 (pCEP4-myc-ACE21 was a gift from Erik Procko: Addgene plasmid # 141185; http://n2t.net/addgene:141185; RRID : Addgene_141185, Addgene, Watertown, MA) into a pCDH lentiviral backbone (System Biosciences, Palo Alto, CA). Vesicular stomatitis virus G glycoprotein (VSV-G) Pseudotyped HIV-derived lentiviral particles were then produced using the pPACKH1 HIV Lentivector Packaging Kit (System Biosciences, Palo Alto, CA) according to the manufacturer’s instructions and used for 293T.hACE2 cell generation. Cells with high ACE2 expression, as validated with flow cytometry, were then isolated *via* fluorescence-activated cell sorting (FACS). hACE2.293T cells used for cytotoxicity analysis were additionally modified with retroviral vectors to express an enhanced green fluorescent protein (GFP) firefly luciferase fusion gene (GFP.ffLuc) (54). GFP-positive cells were sorted and maintained in the appropriate complete growth medium. GFP expression was confirmed through flow cytometric analysis and luciferase expression was confirmed using D-luciferin and quantification of bioluminescence. All cells were maintained in a humidified atmosphere containing 5% CO2 at 37°C.

### Chimeric Antigen Receptor Synthesis

The sequence of H84T-BanLec (32) was synthesized (GeneArt, ThermoFisher Scientific) and subcloned into a pSFG (55) retroviral vector backbone linked to the intracellular domains of 4-1BB (CD137) and TCRζ (36). More specifically, the lectin is linked to a CD8α hinge and transmembrane domain, CD137 intracellular domain, and the intracellular domain of TCRζ.(H84T-BanLec.4-1BB.ζ). Transgenic sequence fidelity was validated by Sanger sequencing (Johns Hopkins Genetic Resources Core Facility).

### Generation of CAR-NK Cells

Peripheral blood mononuclear cells were isolated from healthy donor leucopaks (Anne Arundel Medical Blood Donor Center, Annapolis, MD). T cells were then depleted with CD3-microbeads (Militenyi Biotec, Cologne, Germany). CD3+ cell depletion was verified with flow cytometry using Phycoerythrin (PE)-conjugated anti CD3 (clone: HIT3a, BD Biosciences, Franklin Lakes, NJ) and Brilliant Violet (BV)421- conjugated anti-CD56 (clone: HCD56, BioLegend, San Diego, CA) antibodies (**Figure S2**). CD3-depleted peripheral blood mononuclear cells were stimulated on day 0 with lethally irradiated K562 feeder cells expressing membrane bound IL15 and 4-1BB ligand (45) at a 1:1 ratio. Cells were maintained in SGCM media (CellGenix, Freiburg, Germany) with 10% FBS and 2 mMol glutaMAX (ThermoFisher) supplemented with recombinant human interleukin (IL)-2 (200 IU/mL, pre-clinical biorepository, National Cancer Institute). Retroviruses carrying our vectors were produced by transfecting 293T cells (ATCC, Manassas VA) with GeneJuice transfection reagent (Millipore-Sigma) and 3.75 μg of the CAR-encoding pSFG vector, 2.5 μg of the pRD114 envelope, and 3.75 μg of Peq-pam-3 plasmid containing the MoMLV gag-pol sequence. Viral vector was harvested from cell supernatant after 48 hours and filtered through 0.45 μm membranes before storage at -80C. NK cell transduction was performed on day 4 of the culture by centrifugation of the retroviral particles onto RetroNectin (Clontech Laboratories, Palo Alto, CA) coated (non-treated) 24-well tissue culture plates at 2000g for 90’ followed by plating of 250,000 NK cells. After 2 days, transduced NK cells were moved off of retronectin into tissue culture treated plates using SCGM media supplemented with 200 IU/mL IL-2.

### Determination of Vector Copy Number

Primer/probe-FAM was designed to the MMLV-derived psi present in pSFG (55) and purchased from ThermoFisher Scientific. RNAseP primer/probe-VIC/TAMRA mix (Applied Biosystems #4403326) was used as comparison. Genomic DNA was isolated from CAR-NK cells and 25 ng used for amplification with TaqMan Universal PCR Mastermix (ThermoFisher) and the above primer/probe mixes on a C1000 Touch Thermal Cycler (Bio-Rad, Hercules, CA). The following amplification conditions were used: 50°C for 2 minutes, 95°C for 10 minutes, 40 cycles of 95°C for 15 seconds, 60°C for 1 minute. No-template, unmodified NK cells and a condition containing only plasmid were used as controls. Vector copy number calculation was performed using the 2^-ΔCt^ method (56).

### SARS-CoV-2 Spike Pseudotyped Viral Assays

SARS-CoV-2 S-protein pseudotyped replication incompetent lentiviral particles were produced by first transfecting 293T with GeneJuice transfection reagent (Millipore-Sigma) and SARS-Related Coronavirus 2, Wuhan-Hu-1 Spike-Pseudotyped Lentiviral Kit (The following reagent was obtained through BEI Resources, NIAID, NIH: SARS-Related Coronavirus 2, Wuhan-Hu-1 Spike-Pseudotyped Lentiviral Kit, NR-52948; individual plasmids (40, 57) at indicated ratios (**Supplemental Table 1**); BEI Resources Repository, Manassas, VA). S-protein pseudotyped viral supernatant was collected 48h after transfection. 1.25x10^4^ hACE2.293T cells were plated on day -1 in black 96-well microplates (Corning, Corning, NY). Parental 293T cells served as a control for nonspecific cell transduction. On Day 0, S-pseudoviral titrations (1:1, 1:5, 1:25, 1:125) were added and the plate was centrifuged at 800g for 30’ at 32°C. Cells were then incubated at 37°C in 5%CO_2_. At 48h post-transduction, the viral-containing supernatant was aspirated and 150ug/ml D-Luciferin containing fresh media added. BL was measured and reactive light units (RLU) determined after subtraction of virus-only background. For co-culture assays containing NK cells and pseudovirus transduced target cells, pseudoviral particles were titrated first on hACE2.293T cells and added to achieve 100-200 maximum RLU. NK cells were added to the hACE2.293T and pseudovirus immediately after centrifugation. BL change was calculated as 100*(baseline BLI – co-culture BLI)/(baseline BLI - background). Baseline BL was measured from wells containing only hACE2.293T and pseudovirus. The assay was performed in experimental triplicate per donor.

### Flow Cytometry

ACE2 expression on 293T cells was validated with flow cytometric analysis, utilizing staining with Alexa Fluor 647 conjugated anti-ACE2 Ab (Clone # 535919; R&D Systems, Minneapolis, MT). CAR expression on the surface of transduced NK cells was evaluated 4 and 14 days post-transduction using primary staining with H84T.BanLec Ab (33) and secondary staining with AlexaFluor647-anti-rabbit F(ab)2 (Jackson ImmunoResearch, West Grove, PA). All samples were acquired on FACSCelesta Cell Analyzer (BD) and analyzed with FlowJo software (v10.6.1). Cell sorting was performed on FACSMelody (BD).

### Binding of Spike Protein to 293T.ACE2

Histidine (His)-tagged recombinant S-proteins: trimera (SPN-C52H9) and D614G trimera (SPN-C52H3) were purchased from ACROBiosystems (Newark, DE). A His-tagged receptor binding domain (RBD) was purchased from R&D Systems (10-500-CV-100, Minneapolis, MN). 293T, hACE2.293T, NK cells, and H84T-BanLec CAR-NK cells were coated with 50ng of recombinant protein, then stained first with His antibody (R&D Systems), followed by PE-anti-mouse F(ab)2 (R&D Systems) and analyzed using flow cytometry.

### Western Blot

NK cells were lysed in RIPA lysis buffer with protease (cOmplete) and phosphatase (PhosSTOP) inhibitor cocktails (Sigma-Aldrich, St. Louis, MO) on ice. Protein quantification was performed using Pierce BCA protein assay kit (cat#23228 and #23224, ThermoFisher) and iMark plate reader (Bio-Rad). Electrophoresis was conducted using Novex WedgeWell 10% Bis-Tris Mini Gels (Thermo Fisher) and protein transferred to polyvinylidene difluoride (PVDF) membrane. Western blot analysis was performed with the following antibodies: mouse anti-human CD247 (clone 1D4; BD Biosciences), mouse anti-human phosphorylated CD247 (pY142, clone K25-407.69; BD Biosciences), and rabbit anti-human H84T.BanLec Ab (33). Membranes were stripped with Restore Western Blot Stripping Buffer (ThermoFisher) and used again for analysis with polyclonal rabbit anti-human glyceraldehyde 3-phosphate dehydrogenase (GAPDH) (Novus Biologicals, Littleton, CO).

### Cytokine Secretion

NK cells were cultured with SARS-CoV-2-pseudovirus transduced hACE2.293T at a 1:1 ratio. Following 48 h of culture, supernatant was harvested and analyzed for interferon (IFN)γ or tumor necrosis factor (TNF)α using enzyme-linked immunosorbent assay (ELISA) kits (Quantikine; R&D Systems) according to the manufacturer’s instruction. Conditions without pseudoviral particles were included as controls.

### Cytotoxicity Assay

NK cells were co-cultured for 48h with hACE2.293T.ffLuc at the indicated effector:target (E/T) ratios. D-luciferin was added to plate and BL measured per well. Mean percentage of specific lysis of triplicate samples was calculated as 100*(spontaneous death–experimental death)/(spontaneous death-background). Spontaneous death was measured with control wells containing only target cells.

### Statistical Analysis

All analyses were performed using GraphPad Prism Software (v9). For comparisons of 2 groups, unpaired two-tailed t tests with Welch correction were used.

## Data Availability Statement

The original contributions presented in the study are included in the article/**Supplementary Material**. Further inquiries can be directed to the corresponding author.

## Author Contributions

CB and IC conceived of the work. CB, DM, and IC developed the methodology. CB, IC, SZ, RR, JS, AM, and JR acquired, analyzed, and interpreted data. CB, IC, and WR wrote and all authors revised the manuscript. All authors contributed to the article and approved the submitted version.

## Conflict of Interest

CB, IC, and DM have pending patent applications describing the use of H84T-BanLec and H84T-BanLec effector cell targeting of SARS-CoV-2.

The remaining authors declare that the research was conducted in the absence of any commercial or financial relationships that could be construed as a potential conflict of interest.

## Publisher’s Note

All claims expressed in this article are solely those of the authors and do not necessarily represent those of their affiliated organizations, or those of the publisher, the editors and the reviewers. Any product that may be evaluated in this article, or claim that may be made by its manufacturer, is not guaranteed or endorsed by the publisher.

## Supplementary Material

Supplementary Table 1 Lists all the plasmids of SARS-Related Coronavirus 2, Wuhan-Hu-1 Spike-Pseudotyped Lentiviral Kit that were used for generation of S-protein pseudotyped lentiviral particles. Plasmid name, type, inserted gene and catalog numbers as well as the microgram ratio used for transfection are listed.

## Abbreviations

ACE2, angiotensin-converting enzyme 2; BanLec, Banana Lectin; BL, bioluminescence; CAR, chimeric antigen receptor; CAR-NK, chimeric antigen receptor natural killer; COVID-19, coronavirus disease 2019; E:T, effector-to-target; ffLuc, firefly luciferase; H84T-BanLec, H84T banana lectin; His, histidine; NK, natural killer; RLU, reactive light units; RBD, receptor binding domain; S-protein, spike protein; SARS-CoV-2, severe acute respiratory syndrome coronavirus 2; UTD, untransduced/unmodified; VCN, vector copy number.
