# Supplementary material for: Glycoprotein Targeted CAR-NK Cells for the Treatment of SARS-CoV-2 Infection
Source: Front Immunol. 2021 Dec 23;12:763460. doi: 10.3389/fimmu.2021.763460 (PMC8732772; doi:10.3389/fimmu.2021.763460)
Supplement: Supplementary Table 1 — Lists all the plasmids of SARS-Related Coronavirus 2, Wuhan-Hu-1 Spike-Pseudotyped Lentiviral Kit that were used for generation of S-protein pseudotyped lentiviral particles. Plasmid name, type, inserted gene and catalog numbers as well as the microgram ratio used for transfection are listed. [file Table_1.docx]

**Supplementary Table**

**Table S1:** Plasmids in SARS-Related Coronavirus 2, Wuhan-Hu-1 Spike-Pseudotyped Lentiviral Kit

| **Plasmid name** | **Plasmid type** | **Insert** | **BEI Resources Catalog Number** | **Microgram-ratio*** |
| --- | --- | --- | --- | --- |
| pHDM.CMV.Spike | Viral Entry Protein | Spike (S) glycoprotein | NR-52514 | 1.7 |
| pHAGE.CMV.ffLuc.IRES.ZSG | Lentiviral Backbone | Firefly Luciferase; ZsGreen | NR-52516 | 5 |
| pHDM.HIV gag-pol | Helper Plasmid | Gag; pol | NR-52517 | 1.1 |
| pHDM.HIV HIV tat1b | Helper Plasmid | Tat1b | NR-52518 | 1.1 |
| pRC.CMV.HIV rev1b | Helper Plasmid | Rev1b | NR-52519 | 1.1 |

*****The ratio of micrograms of each plasmid used during transfection of 293T cells
