# Supplementary material for: Glycoprotein Targeted CAR-NK Cells for the Treatment of SARS-CoV-2 Infection
Source: Front Immunol. 2021 Dec 23;12:763460. doi: 10.3389/fimmu.2021.763460 (PMC8732772; doi:10.3389/fimmu.2021.763460)
Supplement: Supplementary file 2 [file DataSheet_1.pdf]

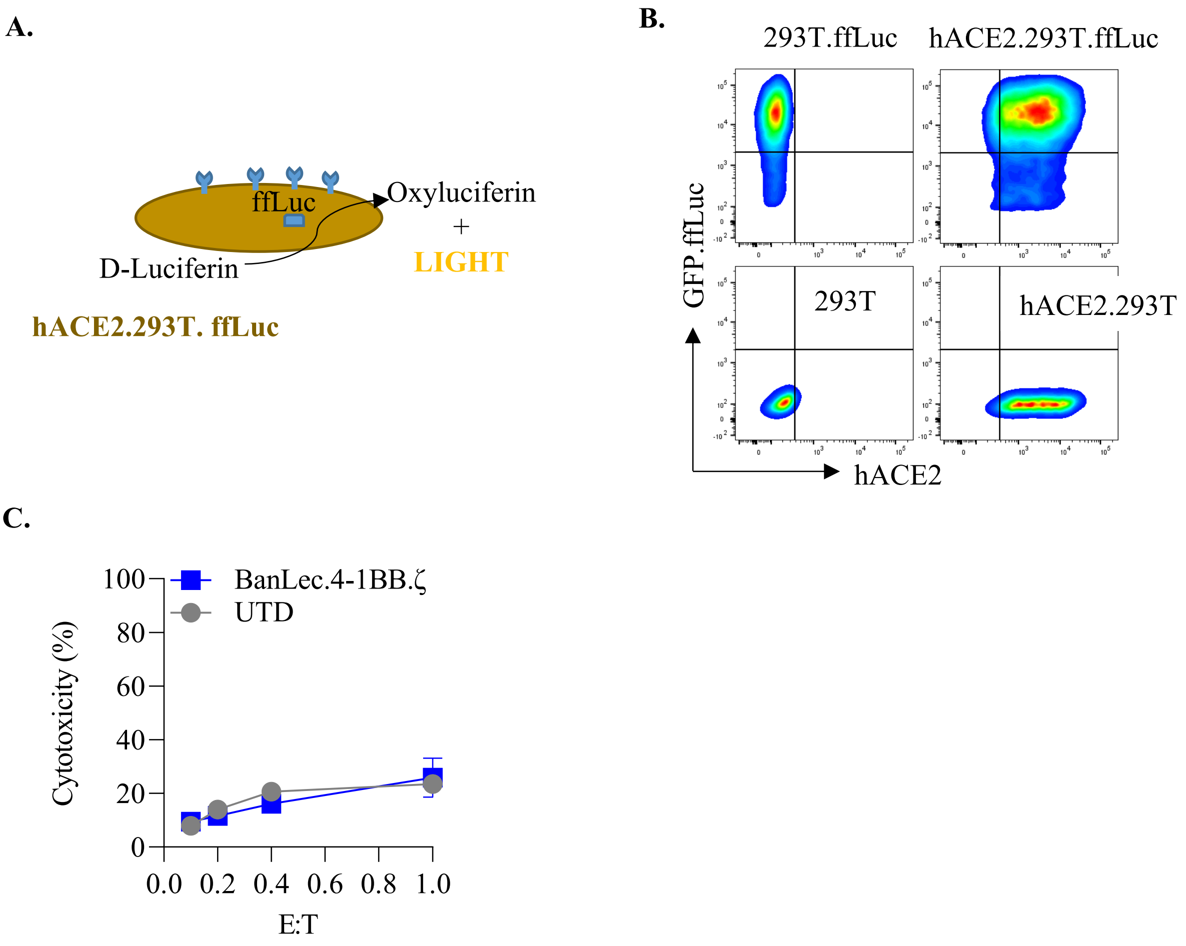

Figure S1. Unmodified and BanLec CAR-NK cells are equally cytotoxic against hACE2.293T targets in the absence of pseudovirus. (**A**) Schema of 293T engineered with both hACE2 and firely Luciferase (ffLuc). (**B**) Representative pseudocolor plots of 293T, hACE2.293T, 293T.ffLuc and hACE2.293T.ffLuc showing ffLuc and hACE2 expression. (**C**) NK cells were co-cultured at indicated E:T ratios with hACE2.293T.ffLuc. Bioluminescence (BL) measured following addition of D-luciferin and compared to control condition without effector cells as an indicator of target cell death (n=6, 2 separate experiments of 3 independent NK cell donors, each experiment performed in triplicate).
