# Supplementary material for: Glycoprotein Targeted CAR-NK Cells for the Treatment of SARS-CoV-2 Infection
Source: Front Immunol. 2021 Dec 23;12:763460. doi: 10.3389/fimmu.2021.763460 (PMC8732772; doi:10.3389/fimmu.2021.763460)
Supplement: Supplementary file 3 [file DataSheet_2.pdf]

A.

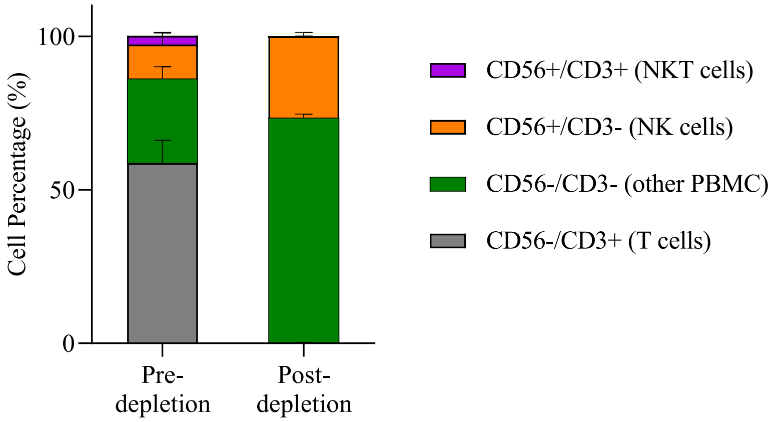

B.

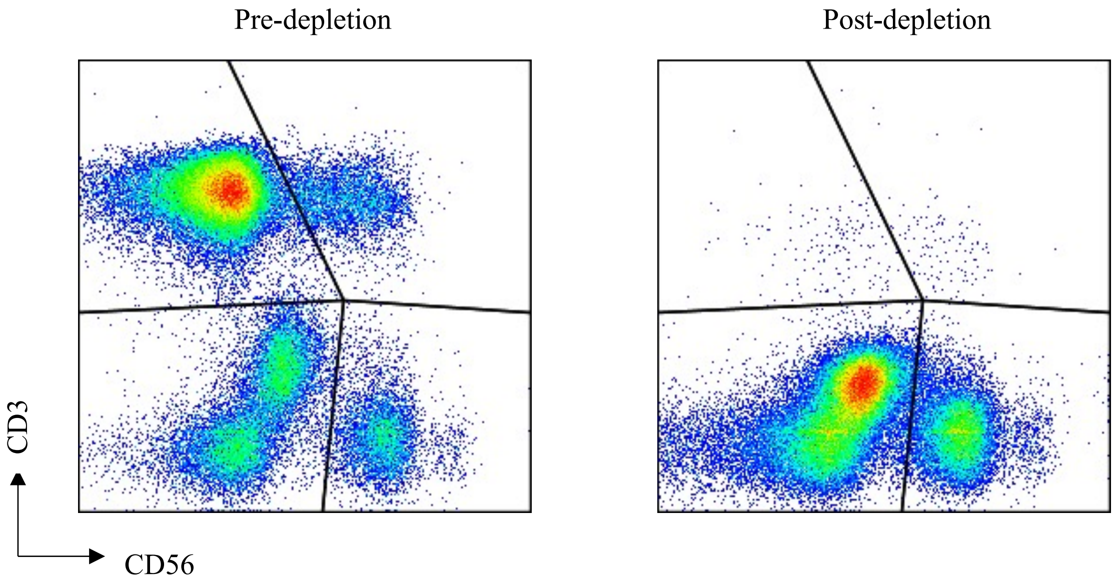

Figure S2. CD3 depletion of peripheral blood mononuclear cells (PBMCs). (**A**) Bar plot depicting the percentage (%) of each PBMC population gated by CD56 and CD3 expression (mean +/- SEM; n=3 donors). Total CD3+ cells (%) Pre-depletion: 61.6 +/- 7.5 vs. Post-depletion: 0.3 +/- 0.1, p=0.0012. (**B**) Representative plots of PBMCs pre- and post-CD3 depletion.
